# Editorial: What’s on the Horizon for asthma treatment?

**DOI:** 10.3389/fphar.2026.1912309

**Published:** 2026-08-14

**Authors:** Luca Novelli, Mauro Maniscalco, Izolde Bouloukaki

**Affiliations:** 1 Pulmonary Medicine Unit, ASST Papa Giovanni XXIII, Bergamo, Italy; 2 Pulmonary Rehabilitation Unit of Telese Terme Institute, Istituti Clinici Scientifici Maugeri IRCCS, Telese Terme, Italy; 3 Department of Advanced Biomedical Sciences, University of Naples “Federico II”, Naples, Italy; 4 Department of Social Medicine, University of Crete, Heraklion, Greece

**Keywords:** airway remodeling, asthma, asthma remission, biomarkers and biologic therapies, precision medicine and genomics, severe asthma

Asthma remains one of the most common chronic respiratory diseases worldwide, affecting hundreds of millions of people and generating a substantial clinical, social, and economic burden (Soriano and Lopez-Campos, 2026). Despite major therapeutic advances over the past two decades, asthma continues to represent an important public health challenge because of its biological complexity, clinical heterogeneity, and variable response to treatment (Agache et al., 2026). Increasing evidence suggests that asthma should not be considered a single disease but rather a group of phenotypes and endotypes driven by different molecular mechanisms (Freeman et al., 2026).

Asthma management is entering a new era. While symptom control remains essential, a deeper understanding of the biological mechanisms underlying the disease is opening new opportunities for more personalized and effective therapies. Future progress in asthma care will depend not only on the development of new therapies but also on improved patient stratification, the identification of predictive biomarkers, and the achievement of disease modification and remission.

The Research Topic *“What’s on the Horizon for Asthma Treatment?”* was conceived to explore these emerging perspectives. The articles included provide a broad overview of recent developments in asthma research, spanning biologic therapies, airway remodeling, metabolomics, microbiome studies, endocrine regulation, epithelial biology, and innovative pharmacological approaches. Collectively, they illustrate the shift from a strategy focused mainly on symptom control toward a precision medicine approach aimed at long-term disease modification.

One of the major themes emerging from this Research Topic is the increasing role of biologic therapies in severe asthma. Beyond their established effectiveness in reducing exacerbations and corticosteroid use, these treatments are increasingly associated with the potential to modify disease progression. In a pilot study, Benfante et al. reported that benralizumab significantly improved inspiratory capacity and eliminated exercise-induced dynamic hyperinflation in patients with severe eosinophilic asthma. These findings link biologic therapy to improved exercise tolerance and daily functioning, extending its impact beyond inflammation alone.

Further evidence supporting the concept of disease modification comes from the study by Lupia et al., who investigated the real-world effectiveness of tezepelumab, a monoclonal antibody targeting thymic stromal lymphopoietin (TSLP), one of the main upstream epithelial alarmins involved in asthma pathogenesis. After 1 year of treatment, patients experienced significant improvements in symptom control, lung function, exacerbation rates, and dependence on oral corticosteroids. In addition, chest computed tomography showed reductions in mucus plugging and markers of airway remodeling, while a considerable proportion of patients achieved complete or partial clinical remission. These findings suggest that targeting upstream inflammatory pathways may influence both clinical outcomes and structural disease changes.

The concept of remission itself has become one of the most important developments in modern asthma care. Ledda et al. addressed this emerging therapeutic goal by evaluating the agreement among currently available international definitions of asthma remission in patients receiving biologic therapies. Their results showed substantial concordance among remission criteria proposed by other authors (Canonica et al., 2025). Furthermore, they confirmed that nearly half of biologically treated patients may achieve complete clinical remission. This analysis reinforces remission as a realistic and measurable target, while highlighting the need for a standardized definition (Eggert et al., 2025). Overall, biologic therapies appear to influence multiple dimensions of severe asthma, including symptoms, physiological impairment, structural changes, and ultimately clinical remission.

Another important area of development is the identification of biomarkers that can improve disease characterization and guide personalized treatment strategies (Maniscalco et al., 2026). Using untargeted metabolomic profiling, Sun et al. identified specific alterations in lipid and amino acid metabolism associated with different asthma phenotypes in children. These metabolic signatures offer a framework for objective disease stratification and support the integration of multi-omics approaches into clinical research.

The growing relevance of systems medicine is also highlighted in the review by Lv et al. on the gut–lung axis. Increasing evidence indicates that the gut microbiota plays an important role in regulating pulmonary immunity through microbial metabolites and immune signaling pathways. Changes in microbial diversity and metabolite production may contribute to allergic inflammation, airway hyperresponsiveness, and immune dysregulation. The authors discuss the therapeutic potential of microbiota-targeted interventions, including probiotics, prebiotics, dietary approaches, and metabolite-based therapies. This perspective supports viewing asthma as a systemic condition shaped by immune, metabolic, and environmental interactions.

Several contributions in this Research Topic also focus on mechanisms involved in treatment response and resistance. You et al. investigated the effects of glucocorticoids on airway epithelial integrity and demonstrated that dexamethasone may induce endoplasmic reticulum stress-related epithelial apoptosis. This dual effect, anti-inflammatory activity alongside potential epithelial damage, may contribute to glucocorticoid resistance and highlights the importance of preserving epithelial integrity.

In addition to inflammation and immunity, endocrine pathways may also play a relevant role in asthma pathobiology. In an interesting perspective article, Bossé introduces the concept of the “testosterone paradox”. Although testosterone appears to protect against asthma and airway inflammation in both epidemiological and experimental studies, it also seems to enhance methacholine responsiveness, a characteristic feature of airway hyperresponsiveness. This paradox highlights the complex interplay among sex hormones, airway smooth muscle function, neural regulation, and immune responses.

Another promising area of investigation involves small molecules and naturally derived compounds capable of targeting both airway remodeling and inflammation. Wang et al. studied the effects of 1,8-cineol (eucalyptol), a naturally occurring monoterpene widely used in respiratory medicine. In experimental models, 1,8-cineol reduced airway hyperresponsiveness, attenuated airway wall thickening and smooth muscle remodeling, decreased inflammatory cell infiltration, and inhibited epithelial–mesenchymal transition through modulation of the NF-κB/COX-2 signaling pathway. Given the limited options targeting airway remodeling, such compounds may complement existing therapies by addressing both inflammation and structural changes.

Overall, the articles included in this Research Topic portray asthma as a multidimensional disease shaped by complex interactions among immune, structural, metabolic, microbial, endocrine, and environmental factors. Several key messages emerge from this Research Topic: biologic therapies are increasingly demonstrating disease-modifying potential, remission is becoming a realistic therapeutic target, biomarkers and multi-omics tools are improving patient stratification, and airway remodeling, epithelial dysfunction and systemic regulatory pathways are gaining recognition as important therapeutic domains. The future of asthma treatment is therefore moving toward an integrated precision medicine model that combines advanced biologic therapies, novel biomarkers, microbiome-based interventions, strategies targeting airway remodeling, and innovative pharmacological approaches. These advances should be viewed as complementary to optimized inhaled therapies, which remain the foundation of asthma management for most patients (Agache et al., 2026). As our understanding of disease heterogeneity continues to improve, the goal will extend beyond symptom control toward disease modification, prevention of progression, and sustained clinical remission.

We sincerely thank all authors, reviewers, and editors who contributed to this Research Topic. We hope that the work presented here will stimulate further multidisciplinary research and foster collaborations aimed at accelerating the development of innovative and personalized therapeutic strategies for patients with asthma worldwide.
